# DNA-Binding Magnetic Nanoreactor Beads for Digital PCR Analysis

**DOI:** 10.1021/acs.analchem.3c01418

**Published:** 2023-08-30

**Authors:** Theresa Heinrich, Susanne Toepfer, Katrin Steinmetzer, Monique Ruettger, Ines Walz, Lea Kanitz, Oliver Lemuth, Stephan Hubold, Friederike Fritsch, Ivan Loncarevic-Barcena, Susanne Klingner, Hartmut T. Bocker, Eugen Ermantraut

**Affiliations:** BLINK AG, Bruesseler Strasse 20, 07747 Jena, Germany

## Abstract

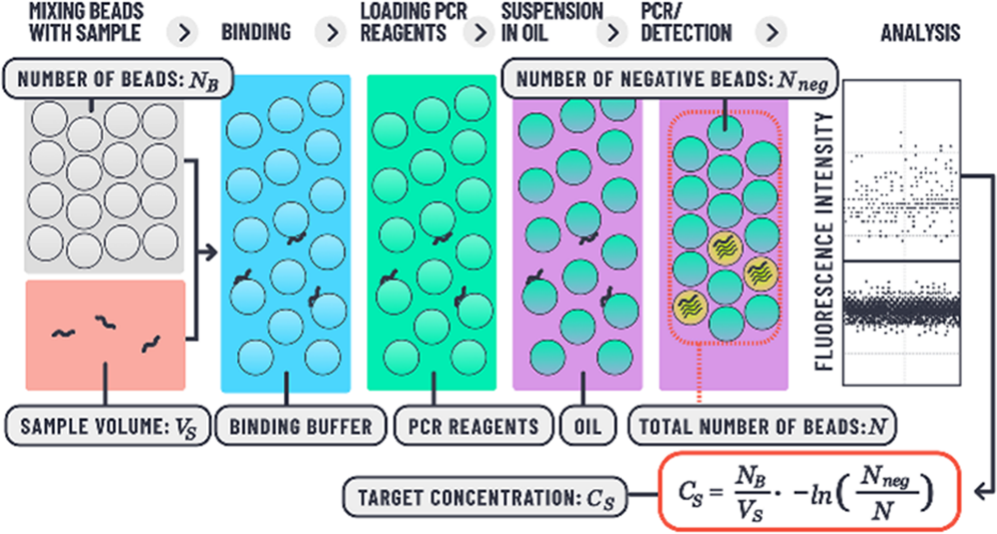

Digital PCR (dPCR) is based on the separation of target amplification reactions into many compartments with randomly distributed template molecules. Here, we present a novel digital PCR format based on DNA binding magnetic nanoreactor beads (mNRBs). Our approach relies on the binding of all nucleic acids present in a sample to the mNRBs, which both provide a high-capacity binding matrix for capturing nucleic acids from a sample and define the space available for PCR amplification by the internal volume of their hydrogel core. Unlike conventional dPCR, this approach does not require a precise determination of the volume of the compartments used but only their number to calculate the number of amplified targets. We present a procedure in which genomic DNA is bound, the nanoreactors are loaded with PCR reagents in an aqueous medium, and amplification and detection are performed in the space provided by the nanoreactor suspended in fluorocarbon oil. mNRBs exhibit a high DNA binding capacity of 1.1 ng DNA/mNRB (95% CI 1.0–1.2) and fast binding kinetics with *k*_a_ = 0.21 s^–1^ (95% CI 0.20–0.23). The dissociation constant *K*_D_ was determined to be 0.0011 μg/μL (95% CI 0.0007–0.0015). A simple disposable chamber plate is used to accommodate the nanoreactor beads in a monolayer formation for rapid thermocycling and fluorescence detection. The performance of the new method was compared with conventional digital droplet PCR and found to be equivalent in terms of the precision and linearity of quantification. In addition, we demonstrated that mNRBs provide quantitative capture and loss-free analysis of nucleic acids contained in samples in different volumes.

Digital PCR (dPCR) assays provide specificity, sensitivity, and precision in nucleic acid analysis.^1^ Compared to bulk PCR methods, dPCR offers the advantage of absolute quantification without the use of a calibration curve.^2^ For this purpose, the amplification mixture is divided into thousands of compartments. This compartmentalization is commonly achieved by creating aqueous microdroplets in an immiscible liquid, e.g., fluorocarbon oil,^3,4^ or by distributing the liquid across some form of nanowells on a solid substrate.^5,6^ These methods require specialized equipment and microfluidic disposables. Quantification of targets in such conventional dPCR is based on the detection of PCR-positive and PCR-negative compartments with a known volume.^7^ Hydrogels have been used in the context of dPCR for encapsulating cells^8^ and for generating beads with immobilized amplicons for post-PCR analysis.^9^ To overcome the inherent need for sophisticated, costly, and error-prone microfluidics, hydrogel beads have been also successfully employed for particle-templated emulsification^10,11^ serving as mechanical templates for the droplet formation in fluorocarbon (FC) oil. The method strongly relies on the ability of the target to penetrate the hydrogel network. Reaching equilibrium between the sample and hydrogel is key to precise target quantitation. Sample utilization is limited, as only a fraction of the liquid with which the hydrogel beads are incubated can be taken up. To facilitate microfluidic-free dPCR and to overcome the limitations of quantitation based on the compartment volume, we followed an alternative concept of distributing targets to compartments. For this purpose, we synthesized nanoreactor beads composed of an agarose core matrix combined with cross-linked chitosan providing for DNA binding. For improved bead handling, we embedded magnetic particles into the hydrogel matrix and thus created magnetic nanoreactor beads (mNRBs) (Figure 1). Chitosan, a linear polysaccharide composed of d-glucosamine and *N*-acetyl-d-glucosamine, has been shown before to be suitable for nucleic acid capture.^12−14^ Because mNRBs are magnetic, sequential process steps requiring the exchange of liquids, such as nucleic acid binding, washing, loading with PCR reagents, and transfer to an oil phase, can be easily carried out. The mNRBs thus combine the requirements for sample handling and partitioning of the reaction mixture for digital PCR in a single bioanalytical reagent.

**Figure 1:**
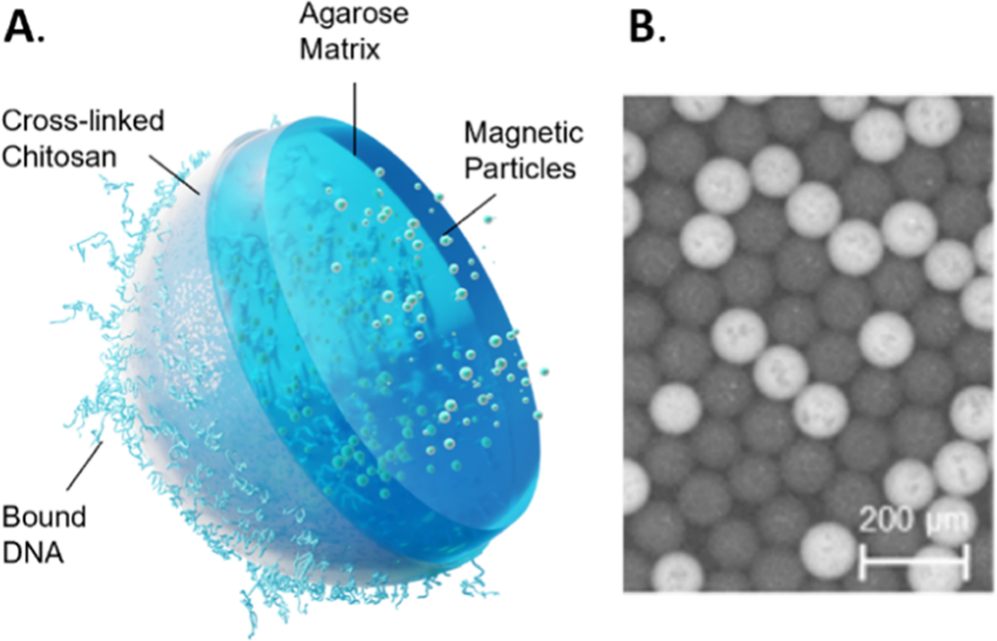
mNRB structure with a hydrogel core composed of non-cross-linked agarose with embedded magnetic particles and a cross-linked chitosan matrix for charge-mediated DNA binding (A), and fluorescence image (B) of processed mNRBs in FC-oil.

We have applied the developed mNRBs to establish a workflow including the steps of DNA binding, washing, PCR reagent loading, transfer to FC oil, thermocycling, and fluorescence imaging as shown in Figure 2. The well plates are processed on a combined thermocycling and fluorescence imaging instrument, the BLINK X (Supporting Information, Figure S1 and Table S1). In conventional dPCR,^2,15^ template is randomly distributed by simply splitting the volume of the master mix across the available amplification compartments. Thus, the target concentration of the sample *C*_s_ is calculated using the following equation
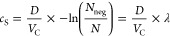
1where *N*_neg_ is the number of PCR-negative compartments, *N* is the total number of compartments, *V*_C_ is the volume of a single droplet or compartment, *D* is the overall dilution factor including dilution steps for sample preparation and master mix setup, and lambda (λ) is the mean number of target molecules per amplification compartment. With mNRBs, random distribution of DNA contained in a sample is achieved by binding the DNA molecules to the beads present in the sample. Thus, DNA can be bound from different sample volumes. Therefore, for calculating the target concentration in a sample, a different equation must be used
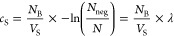
2The mean number of targets per bead λ is calculated using the same Poisson statistics as for droplets, with *N*_neg_ the number of PCR-negative beads and *N* the total number of beads detected. However, no information on the bead volume needs to be taken into consideration for calculating the target concentration in the sample. Instead, the total number of beads applied to the sample *N*_B_ is simply divided by the volume of the sample containing the target *V*_S_ and multiplied by λ. While conventional dPCR provides absolute quantification based on the volume of the compartmentalized sample, the approach presented here is based on target binding, allowing enrichment of targets from different sample volumes and providing target quantification without relying on a precise measure of the volume of the amplification compartments.

**Figure 2:**
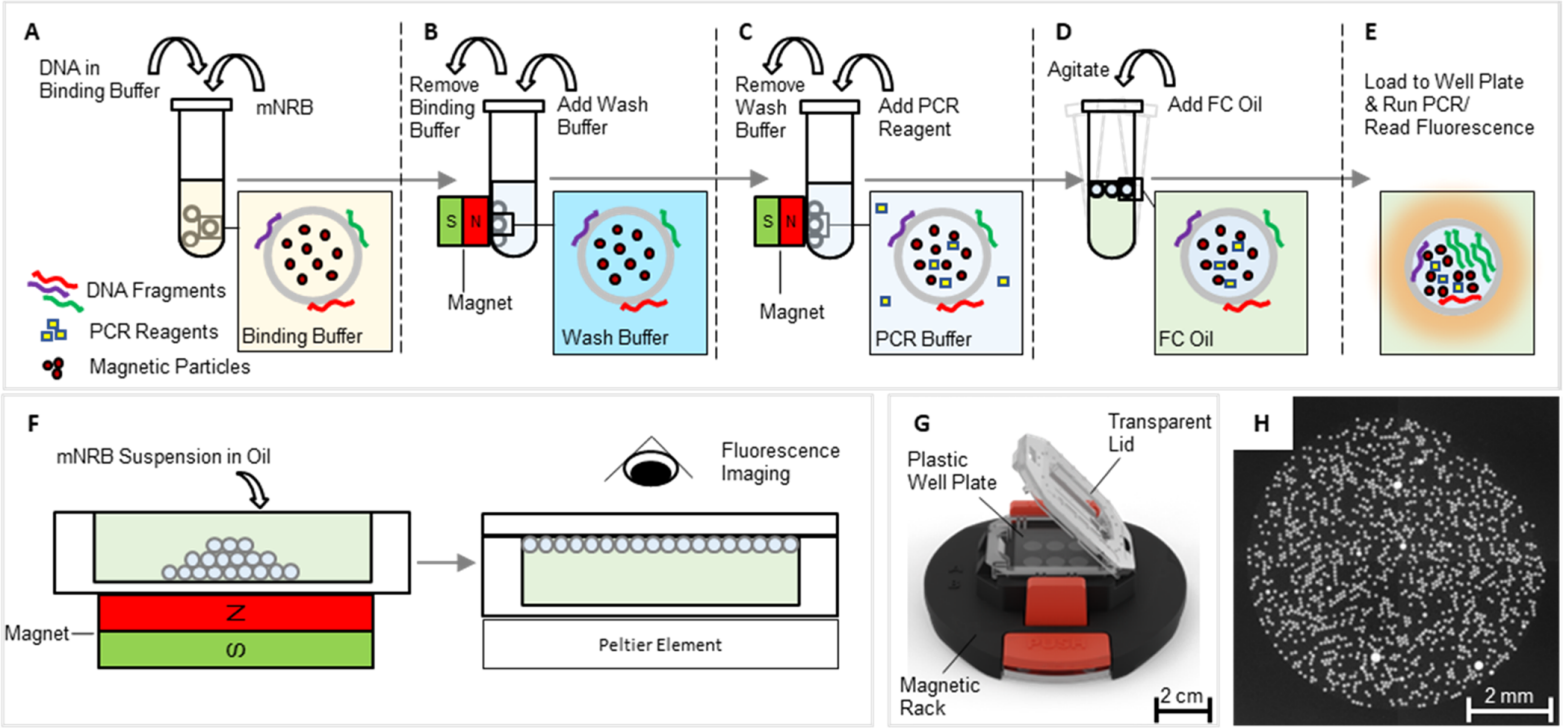
Schematic mNRB workflow. (A) The sample is mixed with binding buffer and incubated with mNRBs. (B) The tube with the beads is placed on a magnetic stand, the binding buffer is removed, and a wash buffer is added. (C) The beads are vortexed and placed back onto the magnetic stand. The wash buffer is replaced with PCR reagents. (D) Following a brief incubation, FC oil is added to the aqueous suspension, and the beads are agitated on a vertical mixer. A bead suspension in FC oil forms with PCR reagents and buffer enclosed within the hydrogel matrix of each mNRB. (E) The suspension is transferred onto a mini-plate and subjected to thermocycling and fluorescence imaging. (F) The process of loading the mini-plate with the bead suspension is shown schematically. (G) The mini-plate is placed on a magnetic rack, and the bead suspension is pipetted into microwells on the plate. Thereby the beads are pulled by the magnet to the bottom of the respective well. After placing a flat cover onto the microwells, the plate is removed from the magnetic rack and placed on the Peltier element of the thermocycling setup. Beads rise to the top, thereby arranging into a self-assembled monolayer. Fluorescence imaging after PCR reveals positive and negative compartments in each microwell on the plate. The shown image represents a well (7 mm diameter) with approximately 3.300 mNRBs.

## Experimental Section

### mNRB Synthesis

A solution containing 0.75% high EEO agarose (Sigma) and magnetic microparticles based on polystyrene (Sigma, 5 μm) at a concentration of 50.000 Particles/μL has been prepared in 100 mM acetate buffer solution pH 4 (Alfa Aesar). Agarose was melted at 99 °C for 30 min and filtered with a 5 μm Cellulose Nitrate Syringe Filter (Whatman) prior to the addition of the magnetic particles. A single emulsion system (Dolomite Microfluidics) was used to generate a monodisperse emulsion consisting of the hydrogel solution and Pico-Surf. To ensure that the composition remained liquid and the magnetic particles did not settle, the sample was kept at 75 °C in a sample reservoir on a hot plate and stirred at 500 rpm. The hydrogel droplets were collected in a tube on ice and incubated for a minimum of 14 h at 4–8 °C to solidify and build stable nanoreactor beads. Thereafter, the bead emulsion was washed two times with an FC volume equal to the bead bed volume, and the remaining Pico-Surf was removed from the nanoreactor bead emulsion by adding a 1*H*,1*H*,2*H*,2*H*-perfluorooctanol (PFO; Sigma) volume equal to the bead bed volume. The tube was vortexed to break the emulsion and centrifuged for 5 s at 2500 g. The aqueous phase with the beads was transferred to a fresh tube. The nanoreactor beads were then passed through a microsieve with a mesh size of 100 μm and filtered at 70 μm in water (Atechnik). A 50% bead suspension was set in 1 mM HCl and equilibrated for 10 min at room temperature. To activate OH groups of the nanoreactor beads, the beads were incubated for 5 min with 124 mM *N*,*N*-carbonyldiimidazole (CDI, Fisher). Thereafter, beads were collected magnetically and washed three times with water (volume of supernatant). Glycine (Fisher) dissolved in water was added directly after CDI activation to the beads to create a 20% bead suspension with 250 mM glycine solution. The solution was incubated for 20 min at 35 rpm on a rotating wheel. Beads were washed five times in a 10-fold volume of water, incubated in water overnight, and washed again two times. To covalently bind chitosan on glycine, a 10% bead solution was set with 0.5% w/v chitosan hydrochloride (Heppe Medical Chitosan) and 9.3 mg/mL *N*-(3-(dimethylamino)propyl)-*N*′-ethyl-carbodiimidehydrochloride (EDC, Sigma) in 50 mM acetate buffer solution pH 4 (Alfa Aesar) and incubated at 4–8 °C while shaking overnight. 1 M Ethylamine hydrochloride (Sigma) was added to a final concentration of 143 mM ethylamine and incubated for 1 h while shaking. Finally, the beads were washed with 50 mM acetate buffer solution at pH 4 and 5× in 1.4 mM HCl in a volume equivalent to the bead volume each. To remove unbound chitosan, a 50% bead suspension was incubated for 10 min at 25 °C at 1.000 rpm in 2 M GuaHCl on a thermomixer and beads were washed at least 10 times with 1.4 mM HCl. The produced mNRBs contain an agarose concentration of 0.7% A9793 and 0.05% Cy3-A9793, an estimated final chitosan concentration of 3 pmol/mNRB based on the concentration of deprotonable groups based on pH titration using a Versa Star VSTAR83 pH meter equipped with a pH microelectrode, and an expected average number of 22 magnetic particles per mNRB. After mNRB production and chemical modification, samples of each new bead batch are analyzed for bead size, bead count, and fluorescence intensity using defined evaluation procedures. The mNRB used in this study comprised an average volume of 0.446 nL and a mean diameter of 94.8 μm (coefficient of variation = 0.07). For the bead count analysis, 9 replicates with 6 μL of mNRB were emulsified in fluorocarbon oil with 5% Pico-Surf (Sphere Fluidics). Emulsified mNRBs were transferred into one well of a mini-well plate, each prefilled with 960 μL of FC oil containing 0.5% Pico-Surf. The wells of the microplate were imaged by a Blink X instrument for the Cy3-channel. The bead count was determined by image analysis with the image segmentation algorithm and was used to calculate the mNRB concentration in the stock sample of mNRBs. The bead concentration of the mNRBs used in this study is 565 beads/μL. In general, the precision of the bead count protocol was estimated in the context of a method validation study to be 0.05 expressed as the coefficient of variation (CV).

### mNRB Binding Characteristics

Before using mNRBs for dPCR, we investigated the binding characteristics of the mNRBs. For this purpose, we incubated mNRBs with different amounts of DNA and photometrically measured the unbound DNA remaining in the supernatant. Sonicated DNA was used to prevent aggregation of mNRBs as observed for high-molecular-weight DNA at concentrations above 10 ng/μL. The DNA was sonicated on ice (90 × 10 s, amplitude 40%) with an ultrasound sonicator (BRANSON) to yield DNA fragments mainly between 300 and 700 bp. DNA at concentrations between 10 and 400 ng/μL was incubated in a final volume of 100 μL containing 50 mM sodium malonate, pH 2.8, 0.1% PEG 6000, and 13 900 mNRBs per sample with constant mixing at 1.800 rpm. Hence, the amount of DNA per mNRB was in the range of 0.07–2.88 ng/mNRB. The total volume of mNRBs per sample was calculated to be 6.0 μL based on the number of mNRBs and diameter as determined by image analysis (data not shown). Analysis of adsorption kinetics was done by removing the supernatant of mNRBs at defined intervals to assess the amount of unbound DNA. The DNA concentration in the supernatant samples was measured based on absorbance at 260 nm (1 unit of absorbance corresponds to 50 ng/μL dsDNA). To determine the adsorption isotherms, the equilibrium DNA concentrations were determined after 30 min of incubation. Preliminary tests have shown no difference in unbound DNA concentration between 30 and 60 min (data not shown). The Langmuir model (eq 3) was used to fit the experimental data to estimate maximum binding capacity *q*_max_ and dissociation constant *K*_D_
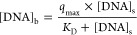
3where [DNA]_s_ is the equilibrium DNA concentration in solution and [DNA]_b_ is the equilibrium DNA concentration adsorbed to the mNRB.

Experimental data for DNA adsorption kinetics were analyzed using eq 4 based on the Langmuir model, with *b_t_* representing the concentration of bound DNA at time *t*, *k*_a_ the adsorption rate constant, *b*_max_ the maximum concentration of DNA bound to the mNRBs, and *C*_0_ the initial DNA concentration in solution.
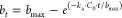
4The nonlinear regression based on the generalized reduced gradient method^16^ was performed using a python script.

### Digital PCR Method Comparison Study

To evaluate the performance of the nanoreactor beads, we conducted a comparative study with mNRBs on the BLINK X Product platform (BLINK, Jena, DE) and droplets on the QX200 system (Bio-Rad). An assay for quantification of the RPP30 target, a single copy gene in human DNA,^17^ was used with both systems. A 10-fold serial dilution series of human DNA containing a sequence target from the RPP30 gene with five dilution levels (V1–V5) was prepared to assess the measuring range and precision of the new assay. Expected target concentrations were set to a range between 25.000 cP (copies)/μL (V1) and 2.5 cP/μL (V5). For dPCR with mNRBs, this corresponds to 0.0172 ng DNA/mNRB for V1. Each sample at each dilution level was tested with a total of 12 replicates for each method.

### Sample Processing Protocol for dPCR with mNRBs

For DNA binding, mNRBs were incubated with a solution containing target DNA and binding buffer (with a final volume of 200 μL and a final concentration of 50 mM sodium malonate, pH 2.8, and 0.1% PEG 6000 in the mixture with 90.400 mNRBs) for 10 min at 30 °C and 1800 rpm. The supernatant was removed from the mNRBs after placing the tube on a magnetic stand followed by washing with 380 μL of a wash buffer (12.5 mM sodium malonate pH 3.0, 0.001% Tergitol 15S9). The wash buffer was removed after placing the beads on the magnetic stand and mNRBs were loaded with PCR reagents. The concentration of the reagents in the final mix was 100 mM tris(hydroxymethyl)-aminomethan, 22 mM potassium chloride, 22 mM ammonium chloride, 3 mM magnesium chloride, 0.2 U/μL Hot Start Taq DNA polymerase (biotechrabbit), 0.2 mM dNTPs (biotechrabbit), 0.1% (w/v) BSA (Sigma), 0.9 μm primers, and 0.25 μM probe (Metabion) with a final pH of 8.6. The beads were incubated with PCR reagents for 5 min in a Thermomixer (Eppendorf) at 30 °C and 1800 rpm. Next, the supernatant was removed from mNRBs on the magnetic rack. For mNRB emulsification, fluorocarbon oil with 5% Pico-Surf (Sphere Fluidics) was added, and agitation on a Minilys Homogenizer (Bertin Technologies) was performed three times for 5 s at level 2. Excess oil was removed, and fluorocarbon oil with 5% Pico-Surf was added to mNRBs three times to remove microemulsion from the previous emulsification step. 3.5 μL of emulsified mNRBs was transferred to 12 wells in total on two mini-well plates as described in the PCR, Fluorescence Imaging, and Analysis section. The mNRB workflow took 30 min for wet lab sample processing and 50 min for dPCR including detection and analysis.

### Primers and Probes

Primers and a probe specific for human RPP30 gene (forward primer sequence (5′–3′): GCC AAA TTC TGC TCG TTG TTA G; reverse primer sequence (5′–3′): CTT CCC TCA CGG CAT ATA CTT C; probe sequence (5′–3′): Atto488- TCA CCA GCT GGA TGT CCA CAT TCA-BHQ-1) were used to quantify human genomic DNA. The final concentrations for primers and probe were 0.9 and 0.25 μM, respectively.

### DNA Samples

DNA was isolated from human Buffy Coat by using the FlexiGene DNA Kit (Qiagen) according to the manufacturer’s instructions and stored at −20 °C until used. On the day of the experiment, the DNA was thawed and a serial 10-fold dilution was prepared over five steps providing DNA samples that underwent the sample processing protocol with mNRB and QX200.

### PCR, Fluorescence Imaging, and Analysis

For amplification and detection, a BLINK X (BLINK) instrument was used. The instrument features a thermocycling module equipped with a Peltier element and a fluorescence imaging module, providing four channels for fluorescence detection. The system is designed to process mini-plates made of plastics with high thermal conductivity. The plate is positioned on a dedicated magnetic loading rack for filling and aligning mNRB suspensions in a self-assembled monolayer and filled with 960 μL of FC oil containing 0.5% Pico-Surf. To each of the 6 wells of the plate (120 μm depth, 7 mm diameter), the respective reaction-ready emulsified mNRBs are pipetted. The mNRBs are pulled to the bottom of the wells by the magnetic force of the rack. A coverslip is placed into the mini-plate, thereby isolating wells from each other. The mini-plate is closed with a transparent lid containing a gasket at its rim consisting of a thermoplastic elastomer to allow proper melt sealing upon subsequent heat exposure. The closed mini-plate is transferred to the thermocycling module, where it is tightly pressed onto a 40 mm × 40 mm Peltier element for optimal thermal coupling. The module is inserted in the Blink X instrument and thermocycled according to the following temperature profile: initial system priming for 120 s at 80 °C, initial denaturation at 94 °C for 60 s followed by 45 cycles of 94 °C for 2 s and 62 °C for 2 s. End point fluorescence imaging is performed at 30 °C. The mNRBs are imaged on the mini-plate still located in the thermocycling module with the Blink X instrument. For each well position on the mini-plate, 4 images are acquired in each detection channel. Figure S2 (Supporting Information) shows example fluorescence images for different dilution levels and the respective data for the determined fluorescence intensity. Acquired images are saved as raw images and as stitched overview images of the entire plate in bmp format. Data analysis on the BLINK X platform comprises two subsequent analysis steps: image analysis and digital PCR analysis. Image analysis with the image segmentation algorithm utilizes the concept of maximally stable extremal regions^18^ to recognize beads. Based on the identified regions with increased fluorescence signal intensities, circles are fitted that define the outer boundaries of the beads. The representative fluorescence signal intensity of each bead is derived from a fluorescence model, assuming that the beads are spherical. Bead size is directly calculated from the enclosing circles representing outer bead boundaries. Shape and size information is considered for further data processing to detect optical artifacts and apply valid/invalid criteria for each detected mNRB. Digital PCR analysis evaluates the estimated fluorescence signals of beads in the detection channel. As a first step, a fluorescence intensity threshold is determined that distinguishes between PCR-positive and -negative beads. Here, the modality of the fluorescence intensity distribution is determined by fitting a one-component and a two-component Gaussian mixture model (GMM) to the data and selecting the preferred model by comparing their Bayesian information criterion (BIC) scores. In the case of a bimodal distribution, the intensity threshold is the position of the local minimum between the GMM mean values. A Kernel density estimate is applied for curve smoothing and determination of the local minimum. After threshold setting, Poisson statistics evaluates the targets per bead (λ) based on the proportion of PCR-negative beads *N*_neg_ in the total quantity of beads *N*. The final quantitation of the amount of target in the sample *c*_S_ is calculated according to eq 2.

### Sample Processing with the QX200

Digital droplet PCR was performed using the QX200 Droplet Digital PCR System (Bio-Rad) according to the manufacturer’s instructions. The ddPCR Supermix for Probes (no dUTP, Bio-Rad) was mixed with 0.9 μM primers, 0.25 μM probe, and water. 20 μL of PCR reagent mix was supplemented with 5 μL of solution containing target DNA. Droplets were generated using the QX200 Droplet Generator with DG8 cartridges and gaskets (Bio-Rad), transferred to a 96-well plate as recommended by the manufacturer, and sealed using the PX1 PCR plate sealer (Bio-Rad). Thermocycling was performed in a Mastercycler Nexus Gradient (Eppendorf) with an initial denaturation of 10 min at 95 °C, followed by 40 cycles of 30 s at 94 °C and 60 s at 60 °C. After thermal cycling, the plate was held at 98 °C for 10 min followed by cooling to 4 °C. The plate was transferred to the QX200 Droplet Reader for data collection. Results were analyzed using the QX Manager 1.2 Standard Edition software. The QX200 workflow took about 30 min for wet lab sample processing with droplet generation, 120 min PCR run time, and about 60 min detection and analysis with the QX200 device.

### DNA Binding with mNRBs from Different Sample Volumes

DNA binding was tested with increasing binding/sample volume. 171 ng of prequantified DNA was added to different volumes of 148, 214, 330, 532, and 885 μL of binding buffer, each containing approximately 57 000 mNRBs (corresponding to 0.003 ng/mNRB). This resulted in an expected lambda value of ∼1. The incubation temperature was set at 60 °C, and processing was performed according to the sample processing protocol described above in the Sample Processing Protocol for dPCR with mNRB section. Three replicates were run for each sample volume.

## Results and Discussion

**Figure 3:**
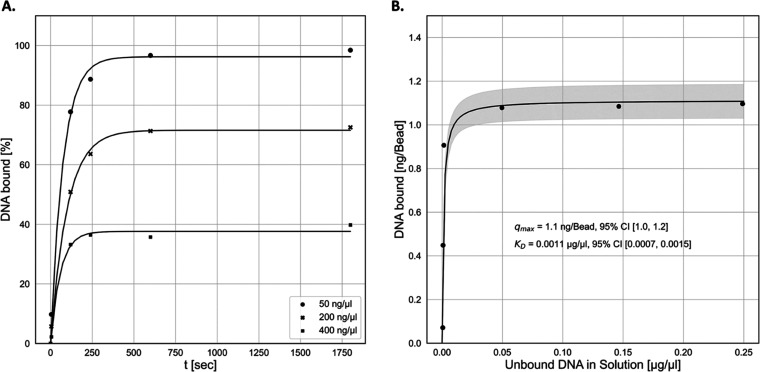
(A) Binding kinetics of genomic DNA to 13.900 mNRBs for initial DNA concentrations of 50 ng/μL (circles), 200 ng/μL (crosses), and 400 ng/μL (squares) in a volume of 100 μL for each sample. The experimental data are indicated by symbols, and the Langmuir model-based fits are shown by the black lines; shown (in %) is the proportion of the amount of DNA present in the sample that was bound to mNRBs. (B) Langmuir isotherm of genomic DNA binding to mNRBs. Experimental data are shown as circles. The input DNA concentrations are 10, 63, 127, 199, 297, and 401 ng/μL, whereas the *X*-axis shows the unbound DNA in solution. The black line represents the Langmuir-based model fit including the 95% confidence interval (gray).

As the new mNRB technology is based on DNA binding, we determined the binding characteristics of mNRBs in advance of a digital PCR method comparison study. The binding capacity and dissociation constant of the mNRBs used in this study were estimated to be 1.1 ng DNA per mNRB (95% CI 1.0–1.2) and 0.0011 μg/μL (95% CI 0.0007–0.0015), respectively. The experimental data and the results of nonlinear regression analysis based on the Langmuir model obtained for the DNA binding capacity of mNRB are shown in Figure 3A. The observed binding capacity of 1.1 ng per single mNRB corresponds to 2.5 mg/mL binding matrix. This conversion allows a comparison with literature values for DNA binding to well-characterized anion exchange resins. The binding capacity determined for mNRBs lies well within the range of 1.3–6.6 mg/mL observed by Tarman et al. for bead-bound plasmid DNA (4 kbp) on anion exchange resin.^19^ However, it is higher than the reported binding capacities between 0.3 mg/mL resin and 0.5 mg/mL resin observed by Cardoso et al.^20^ for plasmid DNA (3.7 kbp) and other reported typical loading capacities of anion exchange resins between 0.5 and 1.0 mg plasmid DNA/mL resin.^21^ While high DNA binding capacity is a desirable feature, the speed of binding is important for practical reasons. The data obtained, shown in Figure 3B, indicate that binding is complete after 10 min under the selected conditions.

The performance of dPCR with mNRB on the Blink X platform was compared with the conventional digital droplet PCR on the QX200 platform. Lambda values obtained with mNRBs are directly derived from the percentage of PCR-negative beads using Poisson statistics. Target concentrations are determined according to eq 2. Each sample was incubated with a total number of *N*_B_ of 90 400 mNRBs. The sample volume of *V*_S_ for each dilution is 16 μL. Due to the significant binding capacity of the mNRBs, complete target capture is assumed for the set experimental conditions with less than 0.02 ng DNA/mNRB, and therefore no adjustments are made for potential target loss during washing and master mix loading. Accordingly, the calculation was performed as follows
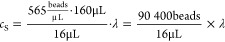
5

**Figure 4:**
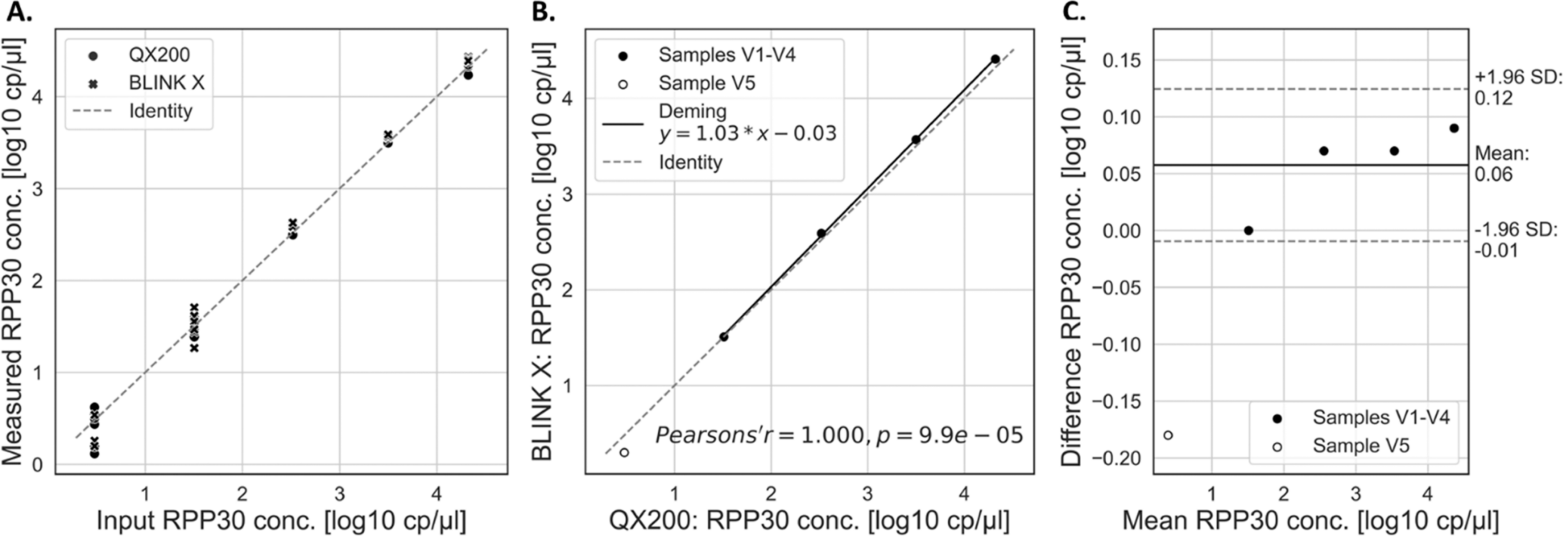
(A) Measurement results obtained with both methods plotted against the expected value. mNRB values are marked with a cross, and QX200 values are marked with a circle; (B) Deming regression plot of mean results obtained for dilutions V1–V4 (samples within measurement range) with droplets and mNRBs on the QX200 and BLINK X, respectively. V5 is not included in the regression analysis because the value is outside of the measurement range. (C) Bland–Altman plot BLINK X mNRB results vs QX200 ddPCR.

The QX200 system determines the target concentration of *c*_s_ in the PCR master mix based on an assumed droplet diameter of 117.5 μm (*V*_C_ = 0.849 nL) according to eq 1. The final target concentration of the QX200 sample is calculated by multiplying the target concentration of the master mix by the total dilution factor D, which results from the ratio of PCR reagent to DNA sample used, which in our case is 5 (5 μL of sample plus 20 μL of PCR reagents mix). Therefore, for the droplets, the target concentration in the sample is calculated as follows
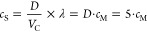
6Lambda values obtained experimentally with mNRBs and droplets can differ significantly. However, when target concentrations calculated for the respective methods are directly compared, as shown in Figure 4A, the results are in good agreement. Based on the measured concentration values, we performed Deming’s regression analysis, which allows the comparison of two measurement methods, both of which may have measurement errors. These results are summarized in Figure 4B. The Deming regression analysis is performed on the log 10 transformed RPP30 concentration values. The slope for the linear regression between BLINK X and QX200 measurements was determined to be 1.03 with a 95% confidence interval (CI) of 0.98–1.07. The estimated intercept is −0.03 with a 95% confidence interval of −0.17 to 0.11. The correlation coefficient Pearson’s r is 1.000, and the *p*-value is 9.9e-05.

As shown in Figure 4C., Bland–Altman analysis reveals a small mean difference of 0.06 log 10 cP/μL between both methods, which may be attributed to droplet size variation in ddPCR using the QX200 system, which has been reported to have a significant effect on the calculated target concentration.^22,23^ In contrast, the size of the mNRBs is not relevant for the target concentration calculation in mNRB technology, but it is based on the number of mNRBs interrogated with the sample. This number is determined for each manufacturing batch of mNRBs according to the protocol described in the Experimental Section. The results for tests performed with mNRBs and ddPCR are summarized in Tables S2 and S3 (Supporting Information), respectively. As shown in Table S2, due to the chosen test design, the sensitivity of the BLINK X method for the highly diluted sample V5 is reduced. Only 4 of 12 replicates provide a nonzero result. This is because not all of the 90.400 mNRBs incubated with the respective sample dilution are transferred to the mini-plate wells for PCR and imaging. The mean number of beads per well (replicate) used here is approximately 3300. The total number of analyzed beads per dilution level is therefore 39.600, which is 44% of all mNRBs applied. Assuming a true V5 sample RPP30 concentration of 3.18 cP/μL (as measured by the QX200), the expected number of targets per test replicate with 3300 beads is only 1.9. Based on Poisson distribution, in this case, detection rate cannot be better than 85%. Thus, to detect V5 reliably above the detection limit, all processed beads would need to be analyzed. The precision of the technical replicates is measured as the standard deviation of log 10 transformed target concentrations. The standard deviations for dilution levels V1–V3 are comparable and very low for both methods. The standard deviation of the BLINK X method for sample V4 is increased, which is caused by the lower number of compartments per replicate compared to the QX200. Overall, the system performs according to the statistically expected values. Performance evaluation in this method comparison study is based on replicate measurements of single sample preparations for each RPP30 target concentration level. For this reason, not all factors that influence precision in a general application are included. In the general case, the expected precision of target concentration estimates with mNRBs depends on the planned total number of beads, the variation of the bead number, the target-to-bead ratio, and the accuracy of pipetting the sample volume. Most effects, e.g., statistical effects of the total number of targets in pipetted sample volume and the random distribution of targets on PCR compartments, are equivalent to other dPCR methods. As in conventional dPCR, precision can be improved by increasing the number of beads and by optimizing the target-to-bead ratio. The main difference in the variance factors is the influence of the bead count variance instead of the compartment size variance. With volume-based sampling, the number of beads is itself subject to a Poisson distribution. However, provision of aliquots with defined numbers of mNRBs by the manufacturing process practically allows elimination of this variance component. Expected trueness of target concentration estimates depends on the amount of targets bound to the beads and on a homogeneous random distribution of targets on beads. Incomplete binding and inhomogeneous distribution of analyte on the beads could lead to an underestimation, which was not observed in the method comparison study. The estimated Langmuir model for binding capacity of beads allows prediction of the amount of target DNA captured by beads. A capture rate of ≥99% is predicted even for the highest RPP30 target concentration V1.

**Figure 5:**
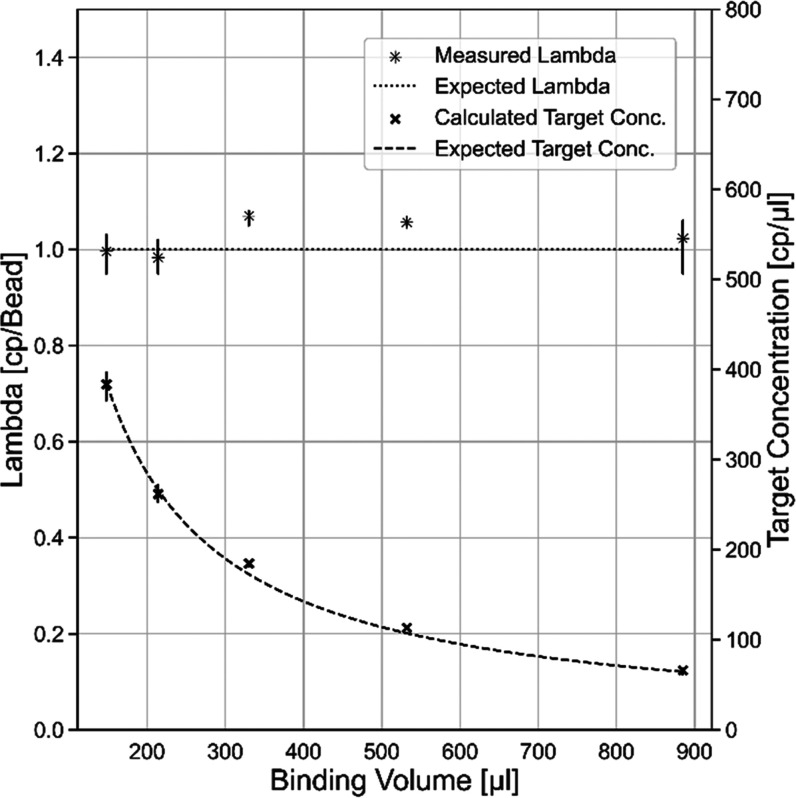
Mean lambda values for different binding volumes are shown as asterisks. The corresponding error bars show the respective minimum and maximum value. Target concentrations calculated based on the respective lambda values and sample volumes are shown as crosses; the minimum and maximum values are shown accordingly. The respective expected lambda values and the calculated expected concentration are shown as dotted and dashed lines, respectively.

To collect the data shown in Figure 4 for the comparison of the new method with the established droplet PCR method with regard to linearity and precision, the sample volume was selected in such a way that the target quantity used could be determined with the reference system within its optimal measuring range. However, this experimental design does not reveal another interesting feature of our method. Since the random distribution of targets to the mNRBs is facilitated by binding, the new approach offers the possibility to process samples with different volumes with the same number of reaction compartments. To demonstrate this feature, we generated samples with different concentrations of DNA by providing the same amount of 171 ng of DNA in different volumes of 148, 214, 330, 532, and 885 μL of binding mix, respectively. The number of mNRBs in each sample was kept constant and adjusted to result in a mean amount of 1 cP of target DNA/mNRB. Three samples were independently processed according to the protocol described in the experimental section. The expected and determined lambda values, as well as the expected and calculated concentration values for each sample are shown in Figure 5.

The data demonstrate that it is possible to determine the concentration of nucleic acid targets with the same number of mNRBs in different sample volumes. The determined concentrations are in good agreement with the expected concentrations across the tested volume range. This opens the possibility to detect rare targets in large sample volumes without having to concentrate the sample or to use a very large number of compartments to analyze the entire sample volume. For example, a QX200 analysis of 750 μL sample volume would need to be split into more than 1.6 million droplets and will require to process 84 individual droplet generation chambers, PCRs, and QX200 read-outs. Conventional volume-based compartmentalization methods are generally limited in their ability to utilize samples with volumes beyond a certain limit. The larger the sample volume, the more compartments are needed to process the sample. With the mNRB method, a solution to this challenge is provided.

## Conclusions

We have presented a new principle for the digital quantification of targets in a sample based on random distribution of targets by binding DNA to the surface of magnetic nanoreactor beads. Using simple tools, mNRBs can be arranged in monolayer formation for PCR amplification and fluorescence imaging. Direct comparison with the ddPCR method shows good agreement of the results obtained with mNRBs with respect to target concentration measurements, while requiring only a fraction of time. Since mNRBs can be produced in large quantities using well-established and cost-effective techniques for the synthesis of monodisperse hydrogel beads, we believe that the cost per assay can be substantially reduced compared to the conventional digital PCR method that relies on complex microfluidic plastic disposables for generating droplets or for providing nanowells with precise volume. With mNRBs, thermocycling and fluorescence detection can be performed with relatively simple instrumentation. Due to their magnetic properties, mNRBs are easy to handle and integration into existing laboratory automation processes appears to be straightforward. Digital PCR with mNRBs is fast, and sample loading can be easily automated due to its similarity to other widely used magnetic bead-based assays and the availability of instrumentation for magnetic bead-based workflows. Unlike other microfluidic-based partitioning methods, the binding approach allows for flexible sample volumes. While it is common today to keep the elution volume small during nucleic acid extraction to be able to transfer the most concentrated sample possible into PCR amplification, the method presented here allows for the use of an eluate that does not have to be limited to a certain volume. This opens the possibility of processing samples with significantly higher initial volumes, thus improving detection sensitivity. Since the calculation of the concentration of a target in the sample is directly related to the number of mNRBs used, it is necessary to determine only the number of mNRBs used for the concentration determination. This can be done by portioning defined amounts of NRBs in the manufacturing process already, e.g., in the form of prealiquoted suspensions or lyophilized materials.

With the current results, we demonstrated that the detection of genomic targets in purified nucleic acid samples is possible with the mNRB method. We anticipate that short DNA fragments can also be detected in the presence of large amounts of genomic DNA as long as the binding capacity of the mNRBs used is not exceeded. This needs to be proven in the context of further investigations.

The mNRB method, in principle, offers the possibility to directly extract nucleic acids from complex biological samples. This will require the identification of optimal buffer conditions to reduce potentially interfering electrostatic interactions of competitor molecules within the sample matrix and to achieve the efficient enrichment and purification of nucleic acids. The development of such integrated processes that allow nucleic acid extraction and target quantification directly from biological samples will be the next step in establishing the mNRB method as an efficient tool for molecular analysis from sample to result.

## Supporting Information Available

BLINK X Instrument, magnetic loading rack, and mini-well plate; specifications of the Blink X instrument; 1D plots of mNRB fluorescence intensity and corresponding fluorescence images; and results from measurements with mNRB and the QX200 reference (PDF)
