# Supplementary material for: DNA-Binding Magnetic Nanoreactor Beads for Digital PCR Analysis
Source: Anal Chem. 2023 Aug 30;95(38):14175–83. doi: 10.1021/acs.analchem.3c01418 (PMC10534990; doi:10.1021/acs.analchem.3c01418)
Supplement: Supplementary file 1 — ac3c01418_si_001.pdf [file ac3c01418_si_001.pdf]

SUPPORTING INFORMATION TO:

"DNA-BINDING MAGNETIC NANOREACTOR BEADS FOR DIGITAL PCR ANALYSIS"

Theresa Heinrich, Susanne Toepfer, Katrin Steinmetzer, Monique Ruettger, Ines Walz, Lea Kanitz, Oliver Lemuth, Stephan Hubold, Friederike Fritsch, Ivan Loncarevic-Barcena, Susanne Klingner, Hartmut T. Bocker and Eugen Ermantraut*

BLINK AG, Bruesseler Strasse 20, 07747 Jena, Germany

*Corresponding author; eugen@blink-dx.com

Table of Contents

Page S2 Figure S1. The BLINK X instrument and mini-well plate
Page S3 Table S1. Specifications of the Blink X instrument
Page S4 Figure S2. 1-D Plots for mNRB fluorescence intensity and corresponding images.
Page S5 Table S2. BLINK X mNRB Results and Table S3. QX200 Droplets Results

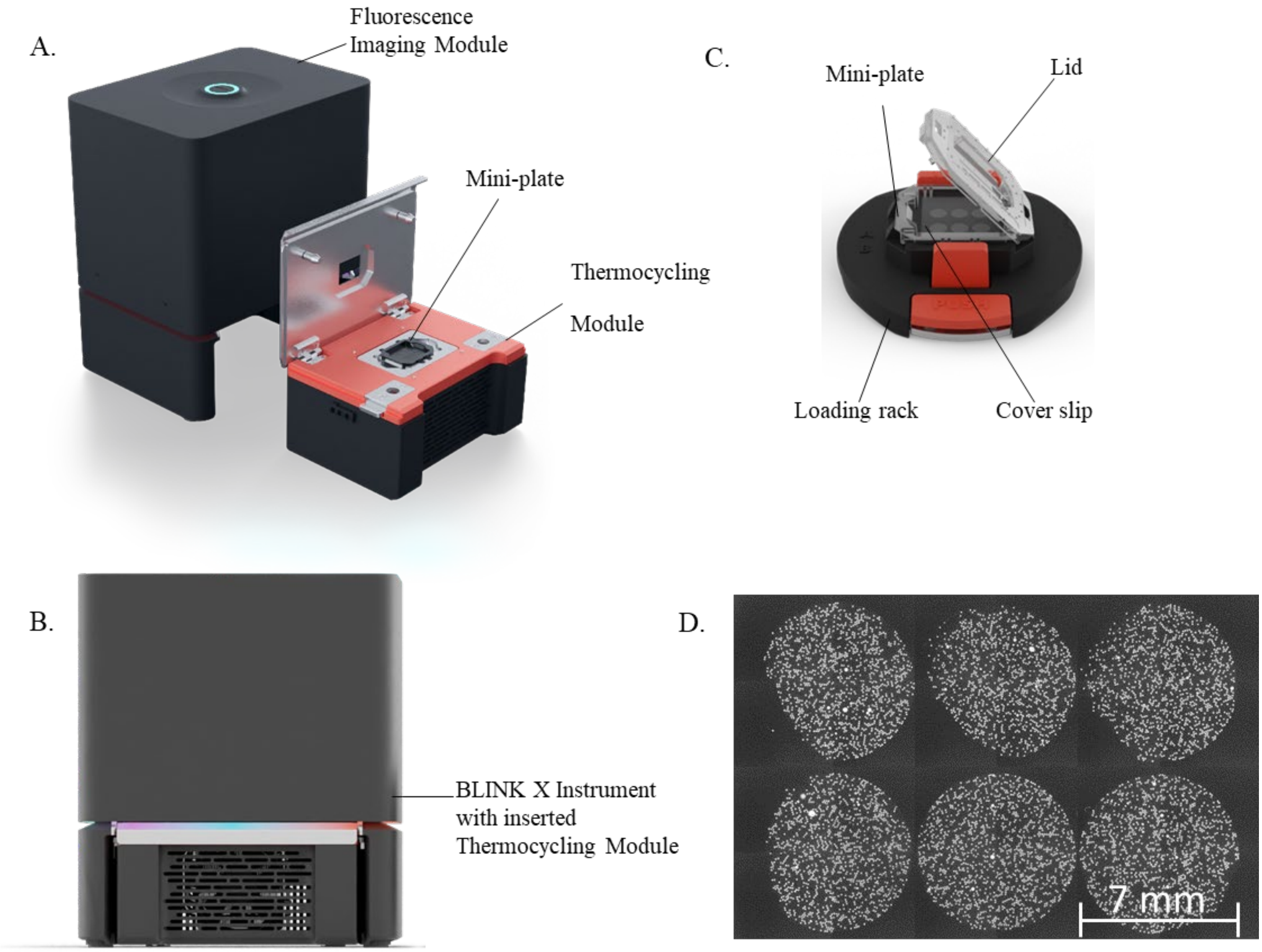

Figure S1. The BLINK X instrument and mini-well plate

(A) Image of BLINK X Instrument with ejected Thermocycling Module with open flap and inserted mini-well plate; Instrument dimensions are 302 mm (height), 258 mm (width) and 288 mm (depth)
(B) Front view of BLINK X instrument with inserted thermocycling module.
(C) Mini-well plate placed on magnetic loading rack, shown with cover slip and lid
(D) representative post-PCR fluorescence image of six wells on mini-well plate.

Table S1. Specifications of the Blink X instrument

**Adjustable Temperature Range**	0°C-99° C (+/-0.5° C), ramps and cycles can be programmed as required				
**Max. Heating or Cooling Rate**	20 K/s resp. 15 K/s				
**Detection Principle**	Epi-fluorescence detection with dedicated LED illumination and filter set per fluorescence channel				
**Fluorescence Channels**		**CH1**	**CH2**	**CH3**	**CH4**
	**LED** peak 100mA-300mA	470- 475	525- 530	620- 625	720- 740
	**Stimulation Filter** in nm	452/45	531/40	628/40	730/39
	**Dichroic Filter** in nm	495	560	660	765
	**Detection Filter** in nm	525/20	585/20	700/50	811/80
	**Compatible Dyes**	Atto488, FAM, FITC	Atto550, Cy3	Atto670Cy5	Atto740, Cy7
**Imaging Area**	32 x 23 mm				
**Single Image Field**	4,8 x 4 mm				

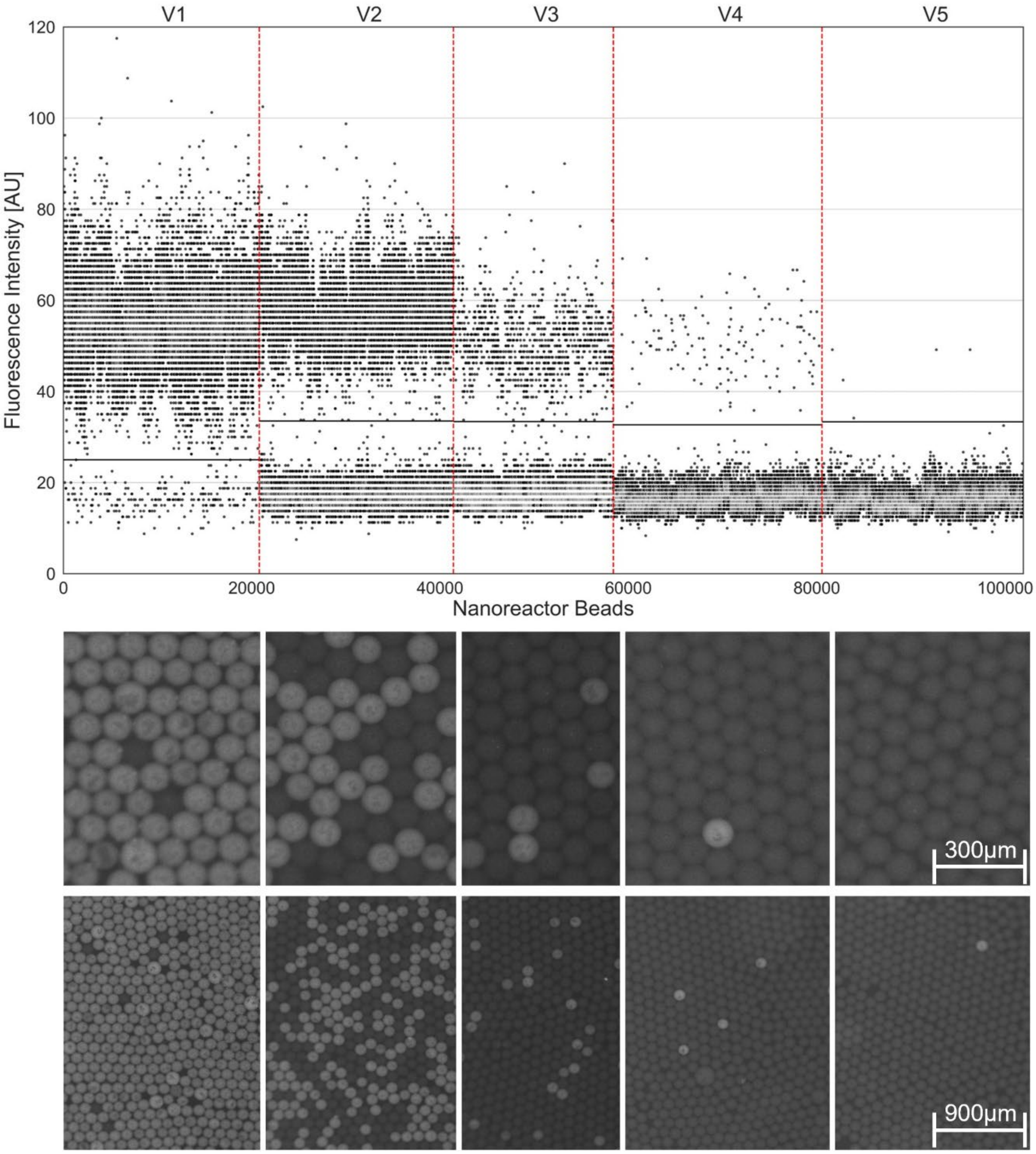

Figure S2. 1-D Plots for mNRB fluorescence intensity and corresponding images. Data shown for five sample dilution levels (V1-V5) and illustrative fluorescence images at different magnifications.

Table S2. BLINK X mNRB Results

Dilution Level	Prop. Neg. Beads Mean [-]	No. Beads *N* Mean	Lambda λ Mean [cp/Bead]	Target Conc. c_S Mean [cp/µL]	Target Conc. c_S Log10 Mean [log10 cp/µL]	Target Conc. cS Log10 Std [log10 cp/µL]
V1	0.01081	3145	4.5386	25643.08	4.41	0.02
V2	0.51874	3371	0.66024	3730.37	3.57	0.01
V3	0.93291	2933	0.06960	393.21	2.59	0.02
V4	0.99418	3500	0.00582	32.89	1.51	0.11
V5*	0.99988	3385	0.00012	2.10**	0.30**	0.16**

* four of twelve replicates yield a non-zero result

** calculated for none-zero results only

Table S3. QX200 Droplets Results

Dilution Level	Prop. Neg. Droplets Mean [-]	No. Droplets *N* Mean	Lambda λ Mean [cp/Droplet]	Target Conc. c_S Mean [cp/µL]	Target Conc. c_S Log10 Mean [log10 cp/µL]	Target Conc. c_S Log10 Std [log10 cp/µL]
V1	0.02971	21696	3.59917	21171.58	4.32	0.05
V2	0.58479	13486	0.53659	3156.39	3.5	0.01
V3	0.94531	16470	0.05625	330.86	2.52	0.02
V4	0.99453	18394	0.00549	32.27	1.51	0.05
V5	0.99946	15342	0.00054	3.18	0.48	0.16
